# The Effect of Model Directionality on Cell-Type-Specific Differential DNA Methylation Analysis

**DOI:** 10.3389/fbinf.2021.792605

**Published:** 2022-01-18

**Authors:** Elior Rahmani, Brandon Jew, Eran Halperin

**Affiliations:** ^1^ Department of Electrical Engineering and Computer Sciences, University of California, Berkeley, Berkeley, CA, United States; ^2^ Bioinformatics Interdepartmental Program, University of California, Los Angeles, Los Angeles, CA, United States; ^3^ Department of Computer Science, University of California, Los Angeles, Los Angeles, CA, United States; ^4^ Department of Computational Medicine, University of California, Los Angeles, Los Angeles, CA, United States; ^5^ Department of Anesthesiology and Perioperative Medicine, David Geffen School of Medicine at UCLA, Los Angeles, CA, United States; ^6^ Department of Human Genetics, University of California, Los Angeles, Los Angeles, CA, United States

**Keywords:** DNA methyaltion, computational biology, differential methylation, statistical analysis, cell-type-specific, EWAS, epigenome-wide association studies

## Abstract

Calling differential methylation at a cell-type level from tissue-level bulk data is a fundamental challenge in genomics that has recently received more attention. These studies most often aim at identifying statistical associations rather than causal effects. However, existing methods typically make an implicit assumption about the direction of effects, and thus far, little to no attention has been given to the fact that this directionality assumption may not hold and can consequently affect statistical power and control for false positives. We demonstrate that misspecification of the model directionality can lead to a drastic decrease in performance and increase in risk of spurious findings in cell-type-specific differential methylation analysis, and we discuss the need to carefully consider model directionality before choosing a statistical method for analysis.

## 1 Introduction

In a typical differential methylation study with DNA methylation data collected from a population, we look for a statistical link between each given methylation site and a condition of interest. Most of the commonly used statistical methods for this task do not aim at identifying statistical links that can be interpreted as causal relations or effects. Nevertheless, the formulation of the underlying model of such methods is most often motivated by possible direct (causal) or indirect (statistical) effects between a condition of interest and a variable of interest, such as methylation. For instance, the commonly used standard linear regression model, in general, cannot be used for inferring causality. However, the underlying model in linear regression posits a certain direction between the variables (i.e., a dependent variable versus independent variables), and flipping the direction of the model can, in general, affect estimation and statistical inference.

In the case of testing for differential methylation, we often model methylation (denote as X) to either be a dependent variable or an independent variable, in which case a condition of interest (denote Y) is set as the dependent variable. We denote these two options for setting the model directionality as X|Y and Y|X, respectively. While we often do not know which one of these two modeling choices better reflects the underlying biology in a given condition, X|Y is arguably a more natural choice in cases where methylation may be *affected* by the condition of interest (either directly or indirectly), and Y|X would be a more natural choice in cases where methylation may *affect* the condition (possibly indirectly).

From a modelling perspective, making a decision about sensible model directionality (and an appropriate method following that directionality) should be study- and context-specific and should depend on the condition under investigation. For example, smoking is known to be statistically associated with changes in DNA methylation (Zeilinger et al., 2013). While it is possible that some of those associations are rising due to genetic variation that affects smoking behaviour (Erzurumluoglu et al., 2020; Xu et al., 2020), it is arguably more likely that most of the observed associations are driven by changes in methylation as a result of smoking; it is therefore more natural to consider the X|Y direction in this case. Another example is the study of differnetial methylation with demographic factors, such as chronological age or ancestry, for which it makes little sense to consider Y|X, as these factors cannot be altered by methylation.

In other cases, investigating Y|X may be more compelling than taking the alternative direction. As an example, consider our recent analysis (Rahmani et al., 2019) of previously studied whole-blood data with rheumatoid arthritis (RA) (Liu et al., 2013). We identified several cell-type level associations with RA, which we then validated using independent sorted metylation data. In particular, we detected three associated CpGs (cg13081526, cg18816397, cg13778567) that are known to be highly heritable: over 50% of the variability of each of these methylation sites is known to be captured by cis-SNPs (Rahmani et al., 2017), reflecting consistency with the possibility that methylation mediates causal genetic effects in RA. This plausible mechanism rationalizes a Y|X directionality. More generally, when direct or indirect causal effects of methylation on a condition are expected, modelling Y|X is a more natural choice.

While the challenge of correctly setting the model diretionality is not specific to one domain, we focus our analysis and discussion on differential DNA methylation. More specifically, we consider the problem of calling differential methylation at a cell-type level from tissue-level bulk data. Learning cell-type-specific effects can be critical for unveiling biological mechanisms (e.g., Claussnitzer et al., 2015), and recent advances in single-cell technologies further emphasized how analysis at the cell-type level can improve our understanding of biology (Buenrostro et al., 2015; Lake et al., 2016; Tirosh et al., 2016a; Tirosh et al., 2016b). As a result, performing cell-type-specific analysis using the abundance of tissue-level bulk data has recently become a primary question of interest in methylation studies (Bauer, 2018; Zheng et al., 2018; Li et al., 2019; Luo et al., 2019; Mendizabal et al., 2019).

Thus far, two main different approaches have been suggested and applied for the identification of differential DNA methylation at a cell-type level from tissue-level bulk data: a regression-based approach (Zheng et al., 2018; Li et al., 2019; Mendizabal et al., 2019) and Tensor Composition Analysis (TCA) (Rahmani et al., 2019). In the regression-based approach, methylation levels are regressed on interaction terms (i.e., multiplicative terms) between cell-type proportions and a condition of interest (i.e., an X|Y model). The effects, estimated by employing standard regression analysis, are then assumed to capture cell-type level variation in methylation, irrespective of possible changes in cell-type proportions between observations. This approach, which has long been suggested and repeatedly established in the context of cell-type-specific differential expression analysis in tissue-level bulk gene expression (Shen-Orr et al., 2010; Westra et al., 2015), was recently proposed in the context of methylation as a method called CellDMC (Zheng et al., 2018); the same idea was also employed for methylation by other groups shortly after (Zheng et al., 2018; Li et al., 2019; Mendizabal et al., 2019).

The second approach, TCA, was recently presented by us. The TCA framework is based on a novel method we developed and applied for modelling cell-type-specific variability in tissue-level bulk data; particularly, we presented it in the context of detecting differential methylation at cell-type-specific resolution (Rahmani et al., 2019). TCA can be applied under either model directionality (i.e., X|Y or Y|X) and can be viewed as a generalized form of regression analysis; for more details, including a comprehensive technical background about both CellDMC and TCA, as well as technical preliminaries for differential methylation analysis at cell-type resolution, see Supplementary Note.

Given that causality is not sought in either of these approaches, the distinction between the two model directionalities may seem semantic or merely a minor, negligible technicality. Yet, as we show here, performing statistical testing under an incorrect model directionality may come with a substantial price in accuracy. Admittedly, it may not always be clear how to properly set the model directionality, and for that reason, it is important to understand the effect and implications of considering an incorrect model directionality. Particularly, the decision on which statistical method to use in the analysis should take into consideration the robustness or sensitivity of the different methods to misspecification of directionality.

## 2 Results

**FIGURE 1:**
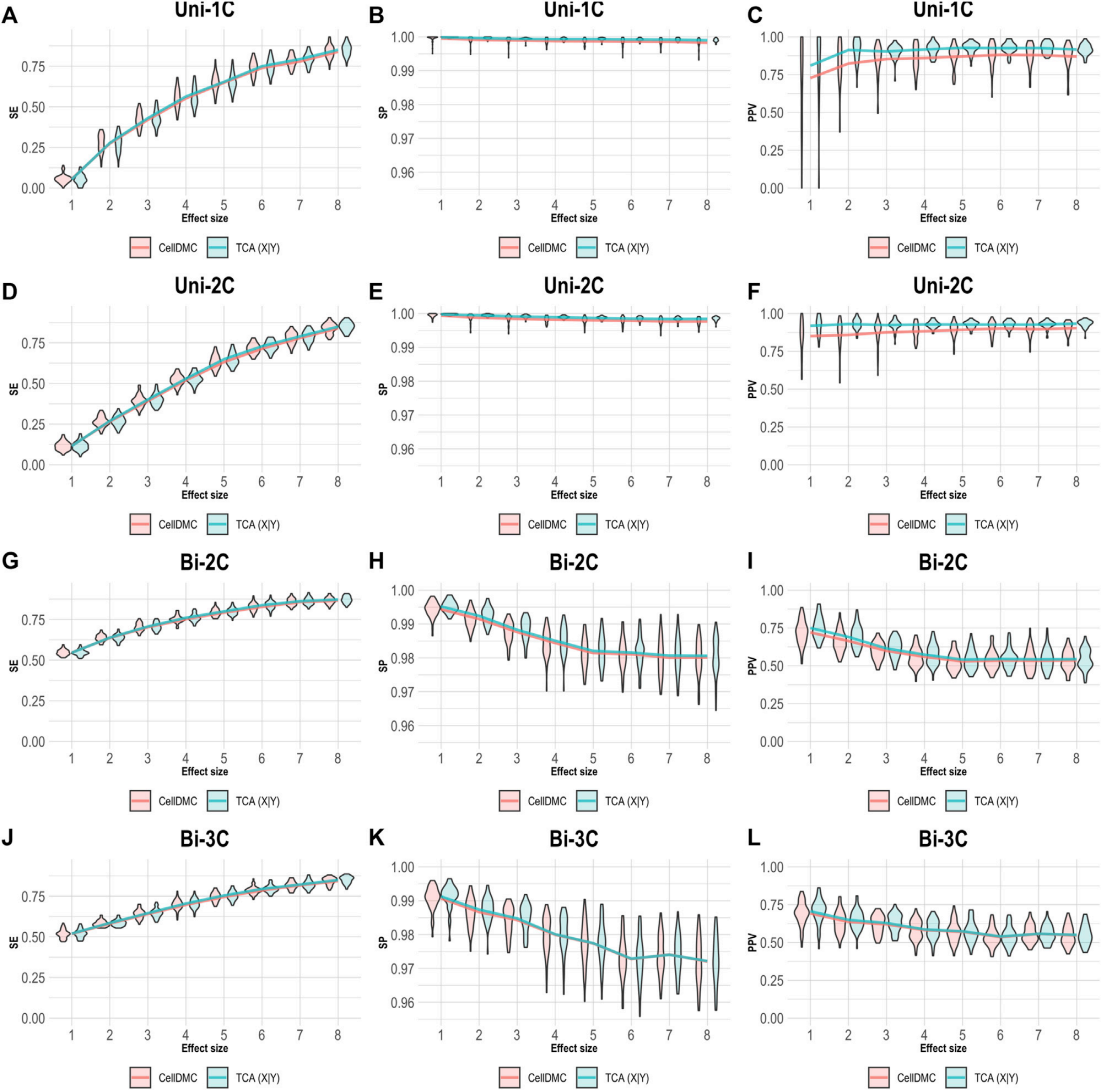
Evaluation of TCA and CellDMC in the case where the phenotype affects methylation (X|Y), while executing both TCA and CellDMC under the correct model directionality (X|Y). **(A–C)** Comparison of the sensitivity (SE), specificity (SP), and precision (positive predictive value; PPV) to detect differentially methylated cell types as a function of the association effect size, under the scenario where a single cell type out of six cell types is altered in cases versus controls (Uni-1C). **(D–F)** as in Uni-1C, only for the scenario where two cell types are altered in the same direction (Uni-2C). **(G–I)** as in Uni-2C, only for the scenario where the cell types are altered in opposite directions (Bi-2C). **(J–L)** as in Bi-2C, only for three cell types (Bi-3C). Results are shown across 50 simulated datasets using violin plots; solid lines represent median values.

In order to understand how misspecification of the model directionality can affect the analysis of cell-type level differential methylation, we conducted a simulation study under several scenarios. We first simulated bulk methylation levels as affected by a phenotype (i.e., following the X|Y direction) and considered cases where there are true associations in one, two, or three cell types, as well as cases where the effect sizes are bidirectional in different cell types (Supplementary Methods). We applied CellDMC and TCA for calling cell-type level differential methylation under the correct direction X|Y, and we measured performance in terms of sensitivity (SE), specificity (SP), and positive predictive value (PPV; also known as the precision), which evaluates the fraction of true positives out of the total number of statistically significant hits reported. The results in Figure 1 demonstrate that both CellDMC and TCA overall provide very high specificity and precision. The slight improvement of TCA over CellDMC is theoretically expected given that the CellDMC model is a degenerate case of the more general TCA model (Supplementary Note).

**FIGURE 2:**
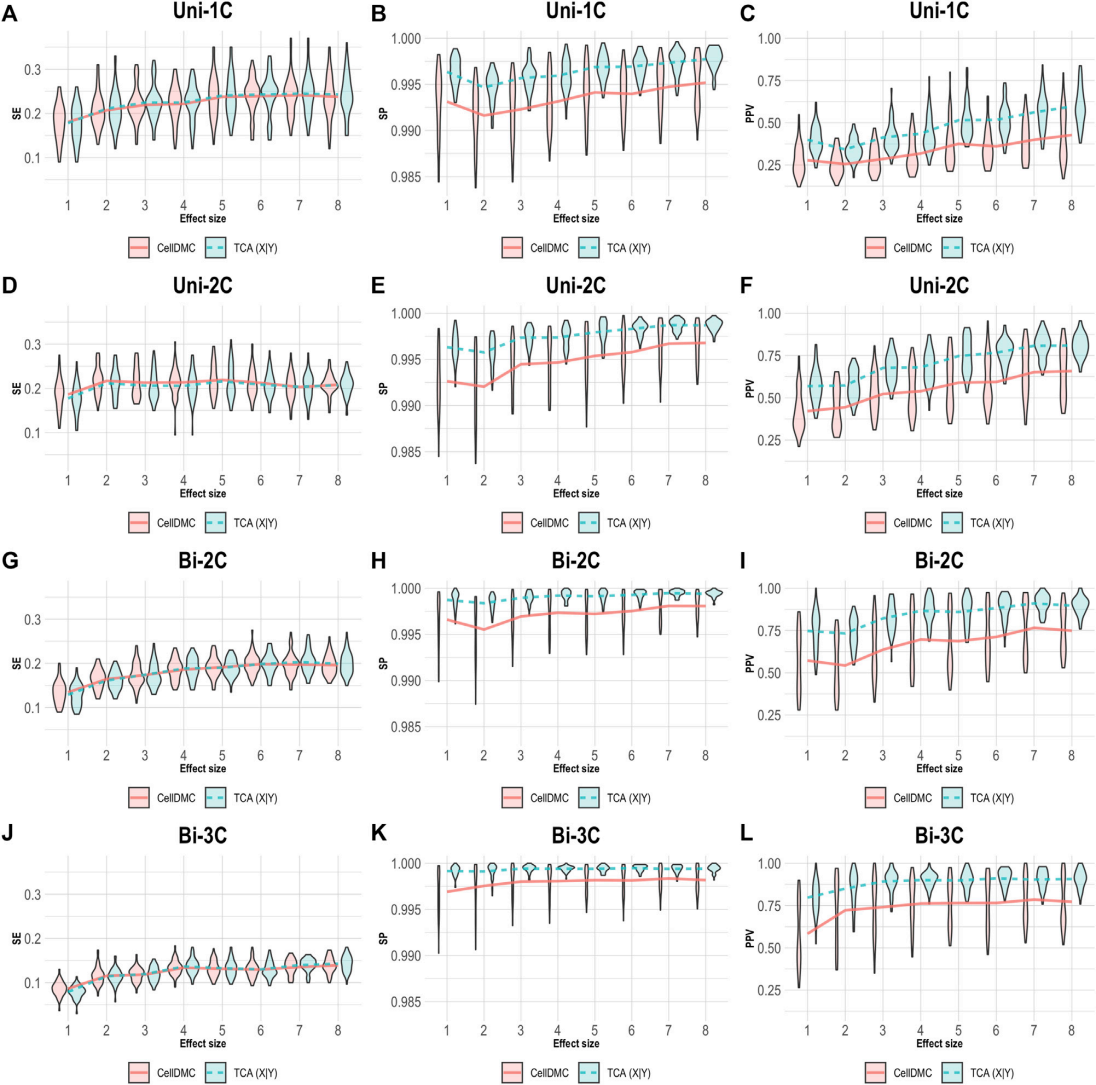
Evaluation of TCA and CellDMC in the case where the phenotype is affected by methylation (Y|X), while executing both TCA and CellDMC under the incorrect model directionality (X|Y). **(A–C)** Comparison of the sensitivity (SE), specificity (SP), and precision (positive predictive value; PPV) to detect differentially methylated cell types as a function of the association effect size, under the scenario where a single cell type out of six cell types is altered in cases versus controls (Uni-1C). **(D–F)** as in Uni-1C, only for the scenario where two cell types are altered in the same direction (Uni-2C). **(G–I)** as in Uni-2C, only for the scenario where the cell types are altered in opposite directions (Bi-2C). **(J–L)** as in Bi-2C, only for three cell types (Bi-3C). Results are shown across 50 simulated datasets using violin plots; solid lines represent median values.

We next simulated phenotypes to be statistically affected by methylation (i.e., setting Y|X as the true model, rather than X|Y; Supplementary Methods) and we evaluated the case where both TCA and CellDMC consider the wrong model directionality. In this case, both methods demonstrate a substantially lower specificity and precision compared to the X|Y simulation, with a more notable decrease for CellDMC (Figure 2).

**FIGURE 3:**
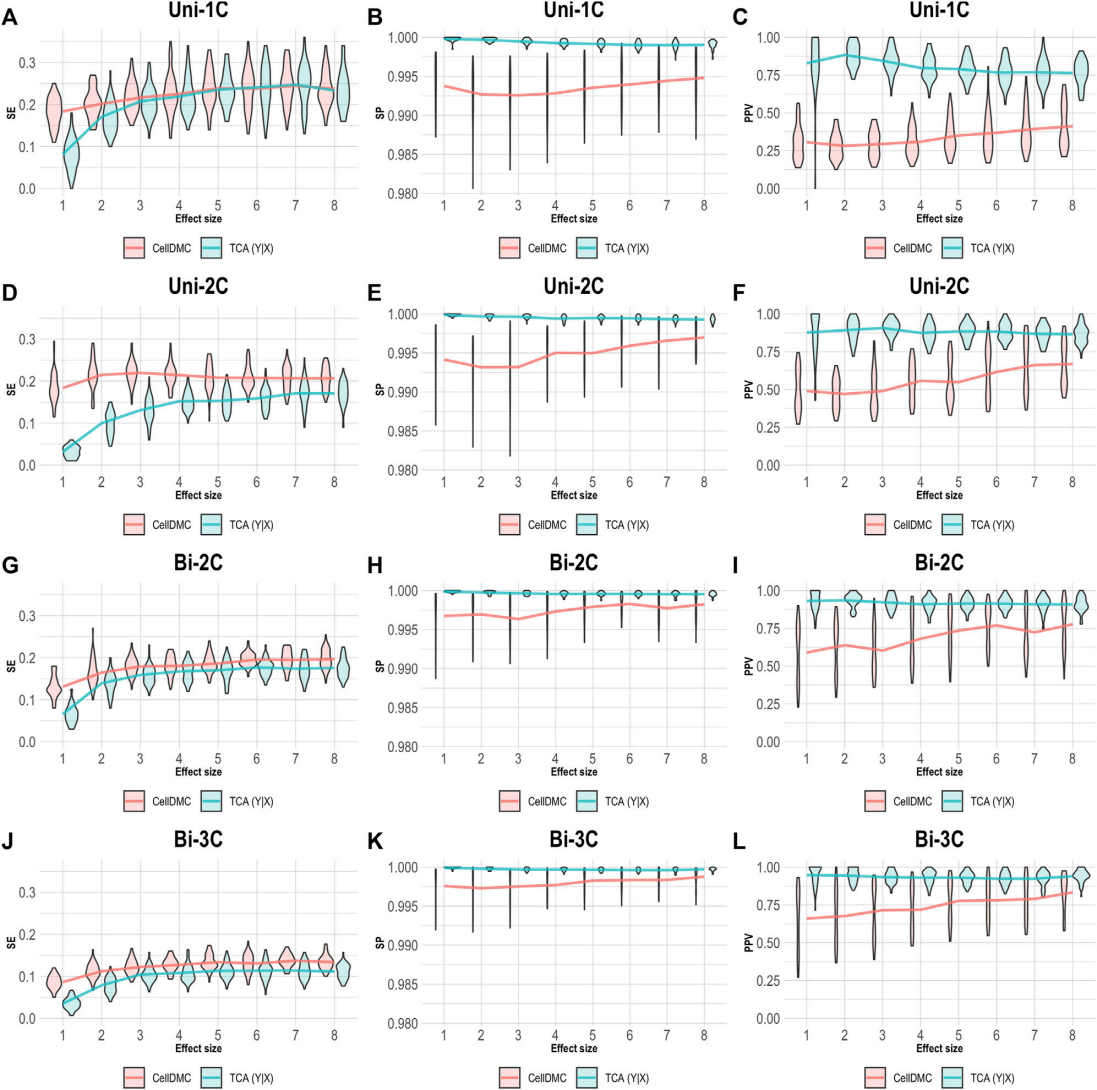
Evaluation of TCA and CellDMC in the case where the phenotype is affected by methylation (Y|X), while executing TCA under the correct model directionality (TCA Y|X) and CellDMC under the incorrect directionality (X|Y). **(A–C)** Comparison of the sensitivity (SE), specificity (SP), and precision (positive predictive value; PPV) to detect differentially methylated cell types as a function of the association effect size, under the scenario where a single cell type out of six cell types is altered in cases versus controls (Uni-1C). **(D–F)** as in Uni-1C, only for the scenario where two cell types are altered in the same direction (Uni-2C). **(G–I)** as in Uni-2C, only for the scenario where the cell types are altered in opposite directions (Bi-2C). **(J–L)** as in Bi-2C, only for three cell types (Bi-3C). Results are shown across 50 simulated datasets using violin plots; solid lines represent median values.

CellDMC is based on regressing methylation levels on interaction terms between cell-type proportions and the condition under test and is therefore limited by design to set the model directionality to be X|Y. TCA, on the other hand, can be applied by setting either model directionality, X|Y or Y|X (Supplementary Note). This allowed us to repeat our simulation of data under Y|X while applying TCA under the correct directionality Y|X. As expected, setting the right model directionality allows TCA to call diferentially expressed methylation sites and cell types at very high precision and specificity (Figure 3). Notably, in this case we observe slightly higher sensitivity for CellDMC compared with TCA, even though the former considers an incorrect model directionality; this is an artifact that stems from an overall inflation in significant statistics reported by CellDMC in this case, which is also evident by the low precision and specificity.

**FIGURE 4:**
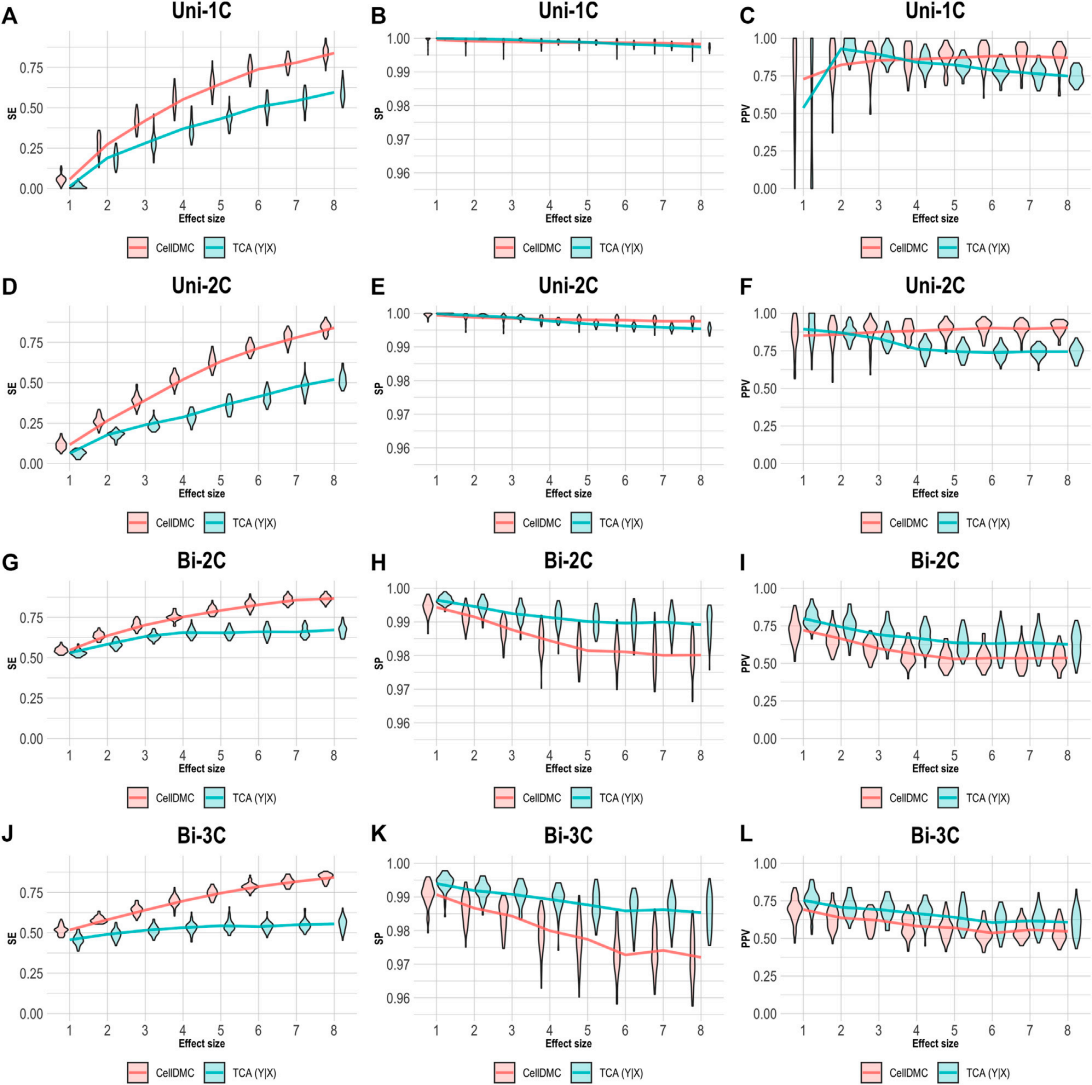
Evaluation of TCA and CellDMC in the case where the phenotype affects methylation (X|Y), while executing TCA under the incorrect model directionality (TCA Y|X) and CellDMC under the correct directionality (X|Y). **(A–C)** Comparison of the sensitivity (SE), specificity (SP), and precision (positive predictive value; PPV) to detect differentially methylated cell types as a function of the association effect size, under the scenario where a single cell type out of six cell types is altered in cases versus controls (Uni-1C). **(D–F)** as in Uni-1C, only for the scenario where two cell types are altered in the same direction (Uni-2C). **(G–I)** as in Uni-2C, only for the scenario where the cell types are altered in opposite directions (Bi-2C). **(J–L)** as in Bi-2C, only for three cell types (Bi-3C). Results are shown across 50 simulated datasets using violin plots; solid lines represent median values.

We further evaluated the case of setting TCA to consider the Y|X directionality on data simulated following X|Y. As before, we applied CellDMC under the (correct) X|Y model directionality in this case, due to the fact that it does not accommodate a Y|X option. As expected, Figure 4 shows that TCA under Y|X present lower precision and specificity compared with the case of simulating data under Y|X (Figure 3). Interestingly, TCA achieves relatively high precision and sensitivity in spite of the misspecification of model directionality. Particularly, TCA avoids false positives better than CellDMC in the scenarios of bidirectional effects; yet, the correctly specified directionality of CellDMC in this case provides better sensitivity than TCA (Figure 4). Overall, our results demonstrate how the relative and absolute performance of different methods can be dramatically affected depending on whether their underlying model correctly specifies the directionality of effects in the data.

Lastly, in order to verify whether our observations on the sensitivity of differential methylation analysis to misspecification of the model directionality are not merely due to the way we simulate data, we further conducted a cell-type level differential methylation analysis with age and sex. Clearly, chronological age and sex cannot be affected by the methylation of an individual, thus rendering X|Y models as as much more natural choice over Y|X models. This setup of an essentially known model directionality, in conjunction with the expected large number of CpGs that are differentially methylated with age and sex (Hannum et al., 2013; Horvath, 2013; Singmann et al., 2015), allows us to evaluate the consistency of real data with our observations from simulations. Particularly, our simulations suggest that in cases where the true underlying model follows X|Y then applying CellDMC and TCA under X|Y yields better precision and specificity compared with setting TCA to consider Y|X (Figures 1, 4). While we do not have a ground truth list of cell-type level differentially methylated CpGs with age and sex, we can evaluate the consistency of each model across studies. A model that tends to report more false positives (and therefore yields lower precision and specificity) is expected to demonstrate lower consistency between the sets of significantly associated CpGs across independent datasets, as false positives are typically not expected to be systematically detected in independent studies.

**FIGURE 5:**
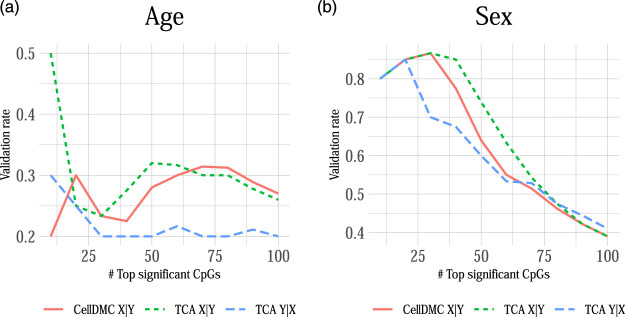
The consistency of calling cell-type level differential methylation with age and sex across two large whole-blood methylation datasets by Liu et al. (2013) and Hannum et al. (2013) (*n* = 687 and *n* = 590, respectively; a set of 129,338 CpGs in both datasets). Presented are the validation rates (Y axes) observed across the two datasets for the analysis with **(A)** age and **(B)** sex using three different methods: CellDMC (which considers X|Y), TCA under X|Y and TCA under Y|X. Validation rate was defined as the fraction of CpGs that were reported in both datasets as the most significant (in terms of lowest *p*-values), using a varying number of the most significant CpGs (X axes); a CpG was counted as reported by both datasets only if it was called as differentially methylated in the same cell type in both datasets.

We applied CellDMC and TCA under both model directionalities to call differential methylation at cell-type level in two independent whole-blood methylation datasets with age and sex information (*n* = 687 and *n* = 590) (Hannum et al., 2013; Liu et al., 2013). In order to evaluate the consistency of each method across datasets, we calculated the method’s validation rate, which we defined as the fraction of associations (i.e., effects in particular CpGs at particular cell types) that were detected by the method in both datasets as most significant (in terms of lowest *p*-values; using a varying number of top significant associations). Figure 5 shows that TCA under Y|X provides lower validation rates compared with CellDMC and TCA under X|Y. In consistency with our simulation study (Figure 1), these results suggest better specificity and precision of the X|Y models in this case. This analysis provides an important complementary evidence for the validity of our observations in simulated data on misspecification of model directionality in cell-type level differential methylation analysis.

## 3 Discussion

Our analysis illustrates how the application of methods under an incorrect model directionality can lead to a drastic decrease in performance, hence increasing the risk of reporting spurious differential methylation results. Model directionality should therefore be carefully considered prior to making a decision on which statistical method to use in a given study.

Considering one direction as more reasonable than the alternative should clearly be context- and condition-dependent. Yet, a sensible decision may not always be straightforward. In the case of differential methylation, based on our results, the level of consistency between TCA and CellDMC may provide a useful evidence as for the true underlying model. Specifically, high consistency in the predicted associations between TCA and CellDMC while applying TCA under the assumption X|Y provides evidence that the assumption X|Y holds (Figures 1, 5). In contrast, limited consistency between the two methods—which is expected to result in more predicted associations for CellDMC over TCA due to lower specificity and precision of CellDMC in this case—can suggest that the assumption Y|X holds; our results show that under Y|X this is expected whether applying TCA under the wrong assumption (i.e., X|Y; Figure 2) or under the correct assumption (i.e., Y|X; Figure 3).

Importantly, the original publications introducing CellDMC (Zheng et al., 2018) and TCA (Rahmani et al., 2019) did not consider both model directionalities in their benchmarking with other methods. In the future, we recommend that the development of new methods and benchmarking of existing methods should be accompanied by a simulation study and benchmarking under both model directionalities presented here. This will allow potential users to be informed about the sensitivity of the different methods to misspecification of the model directionality.

Finally, we bound our discussion to existing statistical methods and models that were previously used for data simulation in the context of cell-type level differential methylation analysis. We acknowledge that our simulation study is limited by its assumptions on the relation and effects between methylation and conditions. In reality, at least in some cases, the true underlying models can be more involved; for example, both methylation and a given condition of interest may be statistically correlated merely due to an unknown third factor (i.e., an unknown confounding factor). In such cases, it may not be clear a priori what would be the meaning and effect of setting different model directionalities as we describe here. We believe that future advances in our understanding of the molecular regulation and roles of methylation in disease and conditions will allow the development of better models, which, in turn, will allow a more accurate evaluation of the sensitivity of different statistical methods to model directionality.

## Data Availability

The original contributions presented in the study are included in the article/Supplementary Material, further inquiries can be directed to the corresponding author.
