# Supplementary material for: The Effect of Model Directionality on Cell-Type-Specific Differential DNA Methylation Analysis
Source: Front Bioinform. 2022 Jan 18;1:792605. doi: 10.3389/fbinf.2021.792605 (PMC9580934; doi:10.3389/fbinf.2021.792605)
Supplement: Supplementary file 1 [file DataSheet1.pdf]

Supplementary Information for:

The Effect of Model Directionality on Cell-Type-Specific Differential DNA Methylation Analysis

Elior Rahmani[1,*], Brandon Jew[2,*], and Eran Halperin[3,4,5,6]

[*]These authors have contributed equally to this work

[1]Department of Electrical Engineering and Computer Sciences, University of California, Berkeley, Berkeley, CA, USA

[2]Bioinformatics Interdepartmental Program, University of California, Los Angeles, Los Angeles, CA, USA

[3]Department of Computer Science, University of California, Los Angeles, Los Angeles, CA, USA

[4]Department of Computational Medicine, University of California, Los Angeles, CA, USA

[5]Department of Anesthesiology and Perioperative Medicine, David Geffen School of Medicine At UCLA, Los Angeles, CA, USA

[6]Department of Human Genetics, University of California, Los Angeles, CA, USA

Contents

1 Technical Background **3**

1.1 Modeling DNA methylation at cell-type resolution: preliminaries 3

1.2 A two-way decomposition for differential analysis with binary phenotypes 5

1.3 Regression with interaction terms as a generalized decomposition for quantitative phenotypes 6

1.4 TCA: a deconvolution-based approach 8

1.5 Relating the TCA model to the interaction model (CellDMC) 11

1.6 TCA: changing the model directionality 12

2 Supplementary Methods **13**

2.1 Software and computational tools 13

2.2 Fast optimization of the TCA model 13

2.3 Simulation study 15

2.4 Cell-type level differential analysis with age and sex 17

1 Technical Background

1.1 Modeling DNA methylation at cell-type resolution: preliminaries In a typical study of methylation collected from a population, we obtain a matrix of measurements $X \in \mathbb{R}^{n \times m}$ of methylation levels in m genomic locations (methylation sites) for n individuals and a condition vector $y \in \mathbb{R}^n$, reflecting a condition or measurement of interest across the individuals in the study.

A common model for evaluating a possible statistical link between a given methylation site j with the condition y can then be formulated as a regression problem in one of two ways:

$$x_{ij} = \beta_j y_i + \epsilon_{ij} \tag{1}$$

$$y_i = \beta_j x_{ij} + \epsilon_{ij} \tag{2}$$

where x_{ij} is the measured methylation at a site j in individual i, y_i is the condition of individual i, β_j is an effect size, and ϵ_{ij} is a residual term for each observation, typically assumed to reflect measurement noise and to be independent and identically distributed across observations. Note that for non-centered data intercept terms should be included.

Equations (1) and (2) reflect two different model directionalities. Specifically, the formulation in Equation (1) sets methylation as the dependent variable and the condition of interest as an independent variable (denote X|Y), and the formulation in Equation (2) considers the opposite model directionality by setting the condition of interest as the dependent variable (denote Y|X).

Population-level studies with methylation predominantly collect data from complex sources and in particular from heterogeneous tissues such as blood; hereafter we limit our discussion to such heterogeneous data. Since methylation can differ between different cell types, a straightforward differential methylation analysis as in Equations (1)-(2) is prone to biases, including decreased power and inflation in the number of false positives[1]. While cell-type-specific data may avoid these biases, the current high costs and practical limitations associated with cell sorting or single-cell biology greatly limits the generation of large-scale datasets. The most widely-used solution to

this challenge is to account for variability in cell-type compositions between samples in the data by adding cell-type compositions as covariates in the regression models in Equations (1)- (2), as follows:

$$x_{ij} = \beta_j y_i + \sum_{h=1}^{k} w_{hi}\mu_{hj} + \epsilon_{ij} \tag{3}$$

$$y_i = \beta_j x_{ij} + \sum_{h=1}^{k} w_{hi}\mu_{hj} + \epsilon_{ij} \tag{4}$$

where μ_{hj} represent the methylation of cell type h in site j and w_{hi} is the proportion of cell-type h in sample i.

Cell-type compositions are rarely measured experimentally, and are therefore typically computationally estimated, most commonly by taking a reference-based approach. The key idea behind this approach recognizes that knowledge of the $\{\mu_{hj}\}$ values in Equation (3) allows a computationally efficient inference of the cell-type proportions (and, similarly, knowledge of $\{w_{hi}\}$ allow inference of μ_{hj}). This concept of decomposing the data into two products (i.e., cell-type proportions and cell-type-specific methylation in this case) has been long studied in the mathematical and statistical literature; most notably, under a non-negative matrix factorization formulation [2]. In the context of genomics, it has been used since the early 2000s to improve interpretation of gene expression mixtures [3,4].

In the context of DNA methylation, such a decomposition was first suggested by Houseman et al. [5] as a reference-based approach for estimating cell-type proportions based on estimates of $\{\mu_{hj}\}$ from methylation in sorted cells; later, this approach was further improved by others in several aspects, such as improving feature selection, collecting reference data from additional populations, or aiming at a better model fit [6–9]. Following the development of these reference-based methods, several unsupervised (reference-free) [10–15] and semi-supervised [16] methods for capturing cell-type composition information have also been developed. Notably, each of those methods can be formulated as a decomposition problem with additional assumptions; for example, the well-known principle component analysis (PCA) essentially performs a decomposition while imposing orthogonality constraints on the decomposition products.

Incorporating the cell-type composition information (denote as W) into the models in Equations (3)-(4) in principle can address the risk of spurious associations due to possible correlations of the condition y with the distribution of cell-type proportions in the data (although in practice, it is typically hard to completely eliminate spurious associations due to various reasons [17]). However, merely including W as covariates conceptually reduces to the case as if individuals in the data were matched for their cell-type proportions; this correction alone does not allow us to probe or recover variation in methylation at a cell-type level (which is expected to be primarily unrelated to changes in cell compositions).

Given the above, for simplicity, consider the hypothetical case of a study with a case-control condition y, such that samples in the study are matched for their cell type proportions (i.e., cases and controls have the same distribution of W and therefore no linear correction for W is required). Biological functions and processes are known to modulate and differ across cell types. Therefore, instead of using the naive tissue-level models in Equations (1)-(2), we would want to consider the following analogue models:

$$z_{hj}^{i} = \beta_{hj} y_i + \epsilon_{hj}^{i} \tag{5}$$

$$y_i = \beta_{hj} z_{hj}^{i} + \epsilon_{hj}^{i} \tag{6}$$

where z_{hj}^{i} is the methylation level at site j in a specific cell-type h for individual i and ϵ_{hj}^{i} represents the cell-type-specific residual term. (As before, we include intercept terms for non-centered data.) The cell-type-specific methylation levels z_{hj}^{i} are not given in a tissue-level study, and our goal is to find ways to approximate the models in Equations (5)-(6).

1.2 A two-way decomposition for differential analysis with binary phenotypes Solving a decomposition problem under the model in Equation (3) assumes that the cell-type-specific levels $\{\mu_{hj}\}$ are shared across all individuals in the data; thus, essentially assuming that all individuals have the exact same methylation and that observed changes in tissue-level methylation are due to differences in cell-type proportions and measurement noise. This assumption, which is unjustified biologically and renders the detection of differential methylation impossible, is inherent in a classical decomposition formulation that factorizes the data into the product of two matrices. The

first relaxation of this assumption in genomics was proposed by Shen-Orr et al. in the context of differential expression analysis [18]. The authors considered the following model for tissue-level expression data:

$$x_{ij} = \sum_{h=1}^{k} w_{hi}\mu_{hj}^{g} + \epsilon_{ij} \tag{7}$$

$$\epsilon_{ij} \sim N(0, \sigma_g^2)$$

Here, x_{ij} corresponds to the expression level of gene j in individual i, w_{hi} corresponds to the fraction of cell type h in individual i, μ_{hj}^{g} for $g \in \{0, 1\}$ represent two possible values (for each of two groups of individuals, such as cases and controls) for the expression of gene j in cell-type h, and ϵ_{ij} is assumed to be normally distributed with a possibly different variance for each group g.

Shen-Orr et al. experimentally measured W and solved Equation (7), which essentially corresponds to solving a supervised version of the decomposition model twice, once for each group g, while fixing W with the measured cell counts. Provided with estimates for $\{\mu_{hj}^{g}\}$, the authors statistically tested for differences between μ_{hj}^{0} and μ_{hj}^{1} for each h, j, which represent differential expression of gene j in cell type h across the two groups of individuals.

The two-way decomposition approach above can in principle be extended to a multi-way decomposition, where a separate decomposition model is fitted for each possible value of a categorical phenotype of interest. However, is it not immediately clear how this approach can be extended to quantitative phenotypes. As we next show, this can be addressed under a regression framework.

1.3 Regression with interaction terms as a generalized decomposition for quantitative phenotypes Westra et al. [19] employed a regression model with interaction terms in the context of differential expression as follows. Keeping the previous notations, the authors considered the following model for tissue-level expression:

$$x_{ij} = \sum_{h=1}^{k} w_{hi}\mu_{hj} + \sum_{h=1}^{k} w_{hi} y_i \gamma_{hj} + \epsilon_{ij} \tag{8}$$

$$\epsilon_{ij} \sim N(0, \sigma^2)$$

where μ_{hj} is the cell-type-specific expression of gene j in cell type h, y_i is the value of individual i for the condition or phenotype of interest, and γ_{hj} is the effect size, specific to gene j, of the interaction (i.e., multiplicative) term between y_i and the proportion of cell type h.

For a binary phenotype $y \in \{0,1\}^n$, it is easy to see that the model in Equation (8) can be rephrased as a two-way decomposition model as in Equation (7):

$$\sum_{h=1}^{k} w_{hi}\mu_{hj} + \sum_{h=1}^{k} w_{hi}y_i\gamma_{hj} + \epsilon_{ij} = \sum_{h=1}^{k} w_{hi}\mu_{hj} + \sum_{h=1}^{k} w_{hi}\mu'_{hj}\mathbb{I}\{y_i = 1\} + \epsilon_{ij} \quad (9)$$

$$\equiv \sum_{h=1}^{k} w_{hi}\mu^{g}_{hj} + \epsilon_{ij} \quad (10)$$

where g in Equation (10) represents the two possible values for the cell-type-specific expression levels corresponding to the two groups encoded by y, exactly as in Equation (7). Of note, the component of variation ϵ_{ij} can be assumed to be identically distributed within a group but not across the two groups.

The above illustrates the motivation for performing cell-type-specific differential analysis with an arbitrary y by testing the coefficients γ_{hj} in the regression model in Equation (8). Clearly, rephrasing the decomposition formulation as a regression problem with interactions not only allows us to consider non-categorical phenotypes, but it also allows a straightforward model fitting and statistical testing by leveraging the well-established tools from standard regression analysis. In practice, fitting the model is done using standard linear regression while adding the multiplication between y and w as covariates. Estimating β is then reduced to solving a standard ordinary least squares (OLS) objective. Additional covariates can be readily included in the model and tested as in standard regression:

$$x_{ij} = \sum_{h=1}^{k} w_{hi}\mu_{hj} + \sum_{h=1}^{k} w_{hi}y_i\gamma_{hj} + \sum_{d=1}^{p} c_{id}\delta_{jd} + \epsilon_{ij} \quad (11)$$

where c_{id} and δ_{jd} are the value of the d-th covariate (out of a total of p covariates) of individual i and its corresponding effect size, which may be specific to feature j.

The interaction model in Equation (11) was recently presented and applied by several groups for

calling differential DNA methylation at cell-type resolution [20–22]. Most notably, it was used in CellDMC by Zheng et al. [20]. Although several works employed this approach, in what follows we consider CellDMC as a representative of this class of work as they are mathematically equivalent and CellDMC appeared first.

1.4 TCA: a deconvolution-based approach While the interaction model in Equation (11) improves upon the classical decomposition model by allowing differences in cell-type-specific profiles between individuals that are coming from different groups (or across a spectrum defined by a quantitative phenotype), it is still limited by the assumption that cell-type-specific levels are constant across individuals when conditioning on the phenotype of interest (whether it is categorical or continuous). Put differently, if we consider a case-control scenario as a simple illustrative example, the regression-based interaction model assumes that all cases (and similarly for controls) share the exact same cell-type-specific levels. The inter-group variation under this model stems only from fixed effects of the phenotype, while ignoring possible effects of additional factors at the cell-type level as well as individual-specific intrinsic variability (i.e., individual-specific biological noise that cannot be explained by any of the observed covariates).

Motivated by these limitations of previous models, we recently introduced the TCA framework, in which we model the assumption that different individuals may demonstrate differences in cell-type-specific profiles owing to multiple factors and individual-specific intrinsic variability [23]. We presented TCA in the context of DNA methylation and applied it for detecting differential methylation at a cell-type resolution.

The TCA model makes the following assumption:

$$Z^i_{hj} = \mu_{hj} + \sum_{d=1}^{p_1} c^{(1)}_{id} \gamma^j_{hd} + \epsilon^i_{hj} \tag{12}$$
$$\epsilon^i_{hj} \sim (0, \sigma^2_{hj})$$

where Z^i_{hj} represents the methylation level of individual i in methylation site j and cell type h (upper case is used as a reminder that in TCA cell-type level methylation is modeled as a random

variable, unlike in previous models), μ_{hj}, σ_{hj} are the population-level mean value and standard deviation for the methylation in site j and cell type h, and $c_{id}^{(1)}, \gamma_{hd}^{j}$ are the value of the d-th covariate (out of a total of p_1 covariates) of individual i and its corresponding effect size that may be specific to site j and cell type h. Note that Equation (12) considers p_1 covariates that may affect methylation in a cell-type-specific manner.

The TCA model further makes the following assumptions about the observed tissue-level data:

$$X_{ij} = \sum_{h=1}^{k} w_{hi} Z_{hj}^{i} + \sum_{d=1}^{p_2} c_{id}^{(2)} \delta_{jd} + \epsilon_{ij} \tag{13}$$

$$\epsilon_{ij} \sim N(0, \tau^2)$$

where X_{ij} represents the observed tissue-level methylation of individual i in site j (upper case is used as a reminder that in TCA the tissue-level data is modeled as a function of multiple random variables, unlike in previous models), ϵ_{ij} is an i.i.d. component of variation, and $c_{id}^{(2)}, \delta_{jd}$ are the value of the d-th covariate (out of a total of p_2 covariates) of individual i and its corresponding effect size that may be specific to site j. Note that Equation (13) considers p_2 covariates that may affect the tissue-level methylation mixtures (i.e., rather than the methylation at the cell-type level; such covariates can be, for example, experimental batches that may affect the mixture levels regardless of cell compositions and the underlying cell-type-specific signals).

The TCA model was originally discussed and evaluated under the Y|X model directionality (see Subsection 1.6), however, in the TCA paper we indicated that the X|Y model can be readily applied under the TCA framework by simply treating y as a cell-type level covariate (see "Applying TCA to epigenetic association studies" in the Methods section of the original TCA paper) [23]. In that case, statistical testing is performed directly within the model in Equation (13).

The TCA model reflects the assumption that the two-dimensional input data (X; individuals by sites) is coming from a three-dimensional underlying structure ($\{z_{hj}^{i}\}$; individuals by sites by cell types). The TCA framework allows us to learn the cell-type-specific levels of an individual from their tissue-level data, thus performing a deconvolution of the observed signals in X into their

underlying signals; of note, we make a distinction between a *deconvolution* that aims at obtaining the complete three-dimensional underlying signals and a *decomposition* that aims at obtaining population-level properties from the data, as in Equation (11). Performing a deconvolution in this case becomes possible by the fact that both the data points and the entries of the underlying three-dimensional tensor are treated as random variables. Consequently, we can evaluate the conditional distribution of the tensor given the observed data and use it for inferring the tensor values of the observed data. Particularly, the TCA estimator is defined as:

$$\hat{z}^i_{hj} = E[Z^i_{hj}|X_{ij} = x_{ij}; \Theta] \tag{14}$$

where Θ represents the parameters in Equations (12)-(13) - these are unknown, however, they can be estimated from the data [23]. Particularly, W is assumed to be known in a typical application of TCA, however, an alternating optimization procedure for re-estimating W from the data in the case where only low-quality estimates of the cell-type proportions are available is possible too [23].

In principle, TCA allows us to replace the cell-type-specific levels in Equations (5)-(6) with the estimates $\{\hat{z}^i_{hj}\}$ and solve simple regression models, either under X|Y or under Y|X. However, as we later demonstrate and discuss in detail, this approach is not always recommended.

Notably, even though the model in Equation (11), as presented in CellDMC, does not make a distinction between cell-type-specific covariates and mixture-level covariates, it can allow so by simply considering interaction terms between the cell-type proportions and the covariates that are assumed to have cell-type-specific effects. Yet, individual-specific intrinsic variability (i.e., the component ϵ^i_{hj} in Equation (12)) is not modeled in CellDMC.

Lastly, in parallel to the introduction of TCA, another group presented essentially the same model as in Equations (12)-(13) [24] and applied it for calling cell-type-specific differential methylation by treating the phenotype of interest as a cell-type-specific covariate (X|Y). The TCA framework, however, is more general: it provides explicit estimates of cell-type-specific methylation profiles for each individual, and as we discuss later, it allows to model either model directionality (i.e., X|Y or Y|X).

1.5 Relating the TCA model to the interaction model (CellDMC) We now establish the relation between TCA and CellDMC, showing that the CellDMC model is a special case of the more general TCA model. For a phenotype of interest y that may affect methylation at the cell-type level (i.e., an X|Y model), assuming no other factors (i.e., covariates) affect methylation for simplicity, the TCA model in Equations (12)-(13) can be formulated as follows:

$$Z^i_{hj} = \mu_{hj} + y_i\gamma^j_h + \epsilon^i_{hj}, \epsilon^i_{hj} \sim N(0, \sigma^2_{hj}) \tag{15}$$

$$X_{ij} = \sum_{h=1}^{k} w_{hi} Z^i_{hj} + \epsilon_{ij}, \epsilon_{ij} \sim N(0, \tau^2) \tag{16}$$

Disregarding variation at the cell-type level across individuals (i.e., assuming $\sigma_{hj} = 0$) we get:

$$z^i_{hj} = \mu_{hj} + y_i\gamma^j_h \tag{17}$$

where z^i_{hj} is now constant given y_i, γ^j_h. (We therefore make a distinction from the notation Z^i_{hj}, which represents a random variable with non-zero variance.) Under this assumption, the model of X can be summarized as follows:

$$x_{ij} = \sum_{h=1}^{k} w_{hi} z_{hi} + \epsilon_{ij} \tag{18}$$

$$= \sum_{h=1}^{k} w_{hi}\mu_{hj} + \sum_{h=1}^{k} w_{hi} y_i \gamma^j_h + \epsilon_{ij} \tag{19}$$

where $\epsilon_{ij} \sim N(0, \tau^2)$, and where, as before, we make a distinction between x_{ij} here and X_{ij} in Equation (16) (where cell-type-specific components are non-trivial random variables). This model is exactly the model in Equation (8), which is the one used in CellDMC.

We conclude that assuming no intrinsic variation at the cell-type level (i.e., setting $\sigma_{hj} = 0$) yields CellDMC as equivalent to the TCA model, which reveals CellDMC as a degenerate case of TCA. Unlike CellDMC, the generality of TCA allows it to absorb and account for intrinsic variability at the cell-type level, and for that reason, TCA is intuitively expected to perform better than CellDMC in cases where intrinsic cell-type variation exists and equally well as CellDMC in cases where intrinsic cell-type variation does not exist or is minimal. For large enough data, we would therefore expect TCA to perform better than CellDMC: when appropriate, it can collapse into the CellDMC

model, and otherwise it can model residual variation that is not modeled by CellDMC; in practice, under the simulation framework in this work, we empirically observe that TCA performs better than CelDMC as long as the data had more than 60 samples (data not shown).

1.6 TCA: changing the model directionality Unlike the regression-based interaction approach in CellDMC, the TCA framework accommodates a Y|X model directionality. Particularly, consider the following model for a given methylation site j:

$$Y_i = \sum_{h=1}^{k} Z_{hj}^{i} \beta_{hj} + e_i \tag{20}$$
$$e_i \sim N(0, \phi^2)$$

where Y_i is the phenotypic level of individual i (upper case is used as a reminder that in TCA the phenotype is a function of multiple random variables, unlike in the previous definition of y) and β_{hj} is the effect size of site j in cell type h on the phenotype.

Fitting the model in Equation (20) within the TCA framework can be done in one of two ways. First, similarly to the X|Y case, cell-type-specific methylation can be inferred following Equation (14), which then allows to employ a standard regression analysis by plugging the cell-type-specific estimates into Equation (20). Second, one can consider the conditional distribution of the phenotype given $\{Z_{hj}^{i}\}$. Since these are random variables with unobserved values, we need to integrated over them; this is made possible using the fact that we observe a realization of X. Specifically, the conditional distribution $Z_{hj}^{i}|X_{ij}$ can be used:

$$\int_{z_1} ... \int_{z_k} Pr\left(Y_i = y_i | \{Z_{hj}^{i} = z_h\}_{h=1}^{k}\right) Pr\left(\{Z_{hj}^{i} = z_h\}_{h=1}^{k} | X_{ij} = x_{ij}\right) dz_1...dz_k \tag{21}$$
$$= \; Pr\left(Y_i = y_i | X_{ij} = x_{ij}\right) \tag{22}$$

Therefore, in this second approach, TCA fits the conditional model $Y_i|X_{ij} = x_{ij}$, which can be expressed in terms of the effect sizes $\{\beta_{hj}\}$, thus allowing to estimate and statistically test them [23]; the full derivations are given in the TCA paper [23].

2 Supplementary Methods

2.1 Software and computational tools We applied TCA using the `TCA` R package version 1.2.0 (available on CRAN); source code is available from github at github.com/cozygene/TCA. For the application of CellDMC, we used the `CellDMC` function in the EpiDISH R package version 2.2.0 (available on Bioconductor) which provides an implementation of CellDMC.

In the application of TCA under X|Y, we used the `tca` function, which provides p-values for the estimated parameters in the model under a marginal conditional test and under a joint test; these are given in the output fields `gammas_hat_pvals` and `gammas_hat_pvals.joint` of the `tca` function. In the application of TCA under Y|X, we used the `tcareg` function, while setting the argument `test` to the requested type of test (e.g., marginal or marginal conditional). Throughout our experiments, we used the fast mode of the `tca` function by setting `vars.mle = FALSE` and the fast mode of the `tcareg` function by setting `fast_mode = TRUE` (see next subsection for details).

Finally, we used the R package `CellTypeSpecificMethylationAnalysis` for our simulation study and for generating the simulation figures. Specifically, the function `parametric_simulations` was used for generating all the simulation figures in this work (while setting `experiment_index=1` for Figures 1 and 4 and setting `experiment_index=3` for Figures 2 and 3). Our differential methylation analysis with age and sex can be reproduced using the script `diff_meth_age_sex.R` from the github repository of the package at github.com/cozygene/CellTypeSpecificMethylationAnalysis.

2.2 Fast optimization of the TCA model We previously applied an alternating maximum-likelihood-based optimization procedure for fitting the TCA model [23]. Learning the mean parameters in the model in equations (12)-(13) given the variance parameters is a convex problem that can be solved efficiently by formulating it as a constrained regression problem, yet, estimating the variance parameters (i.e., given estimates of the means) is a non-convex problem. For that rea-

son, we employed a gradient-based optimization for the variances. This resulted in relatively long runtimes of the function `tca` in the TCA R package, especially for large data such as methylation arrays that include hundreds of thousands of features.

Maximum-likelihood estimation is perhaps the most common approach for fitting statistical models, however, alternatives do exist. Particularly, the generalized method of moments (GMM) allows to estimate model parameters by defining moment conditions, which are essentially sets of equations that are constructed from the model parameters and the data [25]. Given that several assumptions on the moment conditions are met, parameter estimation with proven statistical properties can then be performed by solving efficient quadratic programming problems [25,26].

We applied the GMM technique for a fast estimation of the variances in the TCA model. Particularly, for each methylation site, we estimated the cell-type-specific variance parameters of all cell-types jointly. In order to do so, for each site, we define a set of moment conditions, one per each individual sample in the data. Each such individual-based moment condition formulates an estimator for the variance of the methylation level of the individual in the particular site under consideration. Since the variance of each given individual is a function of both the individual-specific cell-type proportions and the variance parameters of all cell types, these moment conditions can be used to estimate the variance parameters under the GMM framework [25].

We updated the `tca` function in the TCA package to include an argument `vars.mle`, which can set `tca` to use either the original maximum-likelihood estimation procedure for the variances (if set to `TRUE`) or the alternative, GMM-based procedure (if set to `FALSE`). Importantly, setting `vars.mle = FALSE` allows a dramatic reduction in runtime (by almost two orders of magnitude), which results in a computation cost that is almost in par with the efficient regression model suggested in CellDMC (data not shown). This allows us to efficiently conduct large-scale epigenome-wide studies under the $X|Y$ model directionality using the `tca` function.

The TCA framework further allows for statistical testing under a $Y|X$ model directionality; this assumption is implemented in the TCA package within the `tcareg` function. As discussed in the

original TCA paper, there are two approaches to perform statistical testing under Y|X. First, we can use a two-step approach of obtaining estimates for the cell-type-specific levels (using the `tensor` function in the TCA package), which can then be tested for association with the phenotype under a standard regression framework. Second, we can consider a single-step approach of directly using the conditional distribution of the phenotype given the data for statistical inference and testing.

Previously, we implemented only the one-step approach in `tcareg`. In order to streamline the faster statistical testing that is allowed by the two-step approach, we updated the `tcareg` function accordingly to include an argument `fast_mode`, which can set `tcareg` to use either the previously implemented one-step approach (if set to `FALSE`) or the faster two-step approach (if set to `TRUE`).

2.3 Simulation study We designed our simulations based on the simulation setup that was previously suggested by Jing et al. [27] as follows. For each dataset we simulated, we generated tissue-level bulk data for a subset of the methylation sites that are available in the Reinius et al. blood data [28]: 1,000 sites selected at random and an additional set of 333 reference CpGs that are used in the software EpiDISH for reference-based estimation of cell-type proportions [29]. For simulating methylation levels of an individual, we first sampled cell-type-specific methylation levels for the 1,333 sites in each of six major immune cell types (CD4+, CD8+, granulocytes, monocytes, B cells, and natural killer cells) using Beta distributions that we learned from the purified methylation profiles of these cell types in the Reinius et al. data (n=6 for each cell type) [28]. Eventually, we constructed tissue-level methylation values by linearly mixing them according to cell-type proportions that we sampled from a pool of estimates we obtained by applying the reference-based method EpiDISH to large independent whole-blood methylation data [30].

In the simulations under the model X|Y (i.e., methylation is affected by the phenotype), each dataset we generated was consisted of 500 individuals (to reflect a typical sample size in association studies), out of which 250 were cases and 250 controls. For simulating differentially methylated cell-types, we first selected 100 sites and cell types at random (the number of cell types was determined by the specific scenario under consideration as explained later), while requiring the

selected sites to exhibit either low (<0.2) or high (>0.8) average methylation levels in the specific cell-types to be altered (the former were used for simulating hypermethylation in cases and the latter for hypomethylation in cases). Then, we altered the cell-type-specific methylation of cases in the selected sites and cell types based on the following equations:

$$\gamma = \frac{|\mu_1 - \mu_2|}{\sqrt{\frac{\sigma_1^2 + \sigma_2^2}{2}}} \tag{23}$$

$$\sigma_1 = \sqrt{\frac{\mu_1(1-\mu_1)}{\mu_2(1-\mu_2)}}\sigma_2 \tag{24}$$

Here, considering one particular differentially methylated cell type in a given methylation site, γ denotes the effect size and $\{\mu_1, \sigma_1^2\}$, $\{\mu_2, \sigma_2^2\}$ denote sets of the mean and variance of the methylation levels of the particular site and cell type in the cases and controls groups, respectively. Setting μ_1, σ_1 was done using the above equations given a particular effect size under consideration and given the parameters $\{\mu_2, \sigma_2\}$, which were set to the mean and variance of the beta distributions that were estimated based on the Reinius et al. data as explained above. Given $\{\mu_1, \sigma_1^2\}$, these were considered as the mean and variance of a beta distribution from which methylation levels were sampled for cases.

In the simulations under the model Y|X (i.e., methylation affects the phenotype), each dataset we generated was consisted of 500 individuals; these were generated similarly to the X|Y simulated data, with the exception that methylation levels of a specific cell type in a specific site were sampled from a single beta distribution (i.e., the same distribution for all individuals, as opposed to the separate distribution for cases and controls in the X|Y simulations). We selected differentially methylated sites and cell types as performed previously in the X|Y simulations, and then simulated a phenotype for each differentially methylated site j as follows:

$$y_i^j = w_i^T \alpha_j + \sum_{h \in S_j} Z_{hj}^i \beta_{hj} + \epsilon_{ij} \tag{25}$$

$$\alpha_j \sim N(0, 1) \tag{26}$$

$$\epsilon_{ij} \sim N(0, \sigma_j^2) \tag{27}$$

$$\sigma_j^2 = \sum_{h \in S_j} \sigma_{hj}^2 \tag{28}$$

where y_i^j is the phenotypic value of individual i, w_i and α_j are the individual's cell-type proportions and their corresponding effect sizes in site j, respectively, S_j is the set of all differentially methylated cell types in site j, Z_{hj}^i, β_{hj} are the cell-type-specific methylation of individual i in site j and cell type h and the corresponding effect size, respectively, and σ_{hj} is the standard deviation of cell type h in site j.

The rest of the simulated sites that are not differentially methylated were tested against one of the 100 simulated phenotypes (selected at random). Notably, equation (28) allows a meaningful definition of effect sizes by accounting for changes in variability between different methylation sites and cell types. Further, β_{hj} can be either positive or negative under the above formulation, however, in our final evaluations (i.e., in the figures) we consider the absolute value of β_{hj} as the effect size.

In both the X|Y and Y|X experiments, we considered four scenarios that were also suggested elsewhere [27]: unidirectional change in one cell type, unidirectional changes in two cell types, bidirectional changes in two cell types, and bidirectional changes in three cell types. For evaluation metrics, we considered sensitivity, specificity, and precision (positive predictive value ; PPV).

2.4 Cell-type level differential analysis with age and sex We obtained the normalized Illumina 450k data by Liu et al. [31] and by Hannum et al. [30] from the Gene Expression Omnibus (GEO; accession numbers GSE42861 and GSE40279, respectively). We considered only methylation sites from autosomal chromosomes and filtered out non-specific and polymorphic probes [32], as well as low-variance CpGs (variance below 0.001). After removing samples with missing covariate information we were left with 687 samples in the Liu data and 590 in the Hannum data and a total of 129,338 cpgs that were available in both datasets for the analysis.

Our evaluation included three methods: CellDMC, TCA under X|Y and under Y|X (both using marginal conditional tests). Both CellDMC and TCA require cell-type proportions as an input, and since cell counts are not available in the Liu and Hannum datasets, we used a reference-based approach to estimate them. Specifically, we estimated the cell-type proportions of six main

populations of leukocyte cells (granulocytes, monocytes, B cells, natural killer cells, CD4 and CD8 T cells) using EpiDISH [29].

Our analysis accounted for rheumatoid arthritis status, smoking, age (for the analysis with sex), and sex (for the analysis with age) in the Liu data, and for ethnicity, smoking, age (for the analysis with sex), and sex (for the analysis with age) in the Hannum data. In addition, in order to account for technical variation in the data, we also accounted for the top 20 principal components (PCs) that we calculated from the 10,000 least variable CpGs in the data (using the entire 450K data, prior to filtering; these are not expected to exhibit any biological signal but are still affected by global technical variation).

1. Jaffe, A. E. & Irizarry, R. A. Accounting for cellular heterogeneity is critical in epigenome-wide association studies. *Genome Biol* **15**, R31 (2014).

2. Lee, D. D. & Seung, H. S. Learning the parts of objects by non-negative matrix factorization. *Nature* **401**, 788–791 (1999).

3. Lu, P., Nakorchevskiy, A. & Marcotte, E. M. Expression deconvolution: a reinterpretation of dna microarray data reveals dynamic changes in cell populations. *Proceedings of the National Academy of Sciences* **100**, 10370–10375 (2003).

4. Lähdesmäki, H., Dunmire, V., Yli-Harja, O., Zhang, W. *et al.* In silico microdissection of microarray data from heterogeneous cell populations. *BMC bioinformatics* **6**, 1–15 (2005).

5. Houseman, E. A. *et al.* DNA methylation arrays as surrogate measures of cell mixture distribution. *BMC bioinformatics* (2012).

6. Yousefi, P. *et al.* Estimation of blood cellular heterogeneity in newborns and children for epigenome-wide association studies. *Environmental and molecular mutagenesis* **56**, 751–758 (2015).

7. Koestler, D. C. *et al.* Improving cell mixture deconvolution by id entifying optimal dna methylation libraries (idol). *BMC bioinformatics* **17**, 1 (2016).

8. Waite, L. L. *et al.* Estimation of cell-type composition including t and b cell subtypes for whole blood methylation microarray data. *Frontiers in genetics* **7**, 23 (2016).

9. Salas, L. A. *et al.* An optimized library for reference-based deconvolution of whole-blood biospecimens assayed using the illumina humanmethylationepic beadarray. *Genome biology* **19**, 1–14 (2018).

10. Houseman, E. A., Molitor, J. & Marsit, C. J. Reference-free cell mixture adjustments in analysis of dna methylation data. *Bioinformatics* **30**, 1431–1439 (2014).

11. Rahmani, E. *et al.* Sparse pca corrects for cell type heterogeneity in epigenome-wide association studies. *Nature methods* **13**, 443–445 (2016).

12. Houseman, E. A. *et al.* Reference-free deconvolution of dna methylation data and mediation by cell composition effects. *BMC bioinformatics* **17**, 259 (2016).

13. Lutsik, P. *et al.* Medecom: discovery and quantification of latent components of heterogeneous methylomes. *Genome biology* **18**, 55 (2017).

14. Li, Z. & Wu, H. Toast: improving reference-free cell composition estimation by cross-cell type differential analysis. *Genome biology* **20**, 1–17 (2019).

15. Thompson, M., Chen, Z. J., Rahmani, E. & Halperin, E. Confined: distinguishing biological from technical sources of variation by leveraging multiple methylation datasets. *Genome biology* **20**, 1–15 (2019).

16. Rahmani, E. *et al.* Bayescce: a bayesian framework for estimating cell-type composition from dna methylation without the need for methylation reference. *Genome biology* **19**, 141 (2018).

17. Rahmani, E. *et al.* Correcting for cell-type heterogeneity in dna methylation: a comprehensive evaluation. *Nature methods* **14**, 218 (2017).

18. Shen-Orr, S. S. *et al.* Cell type–specific gene expression differences in complex tissues. *Nature methods* **7**, 287 (2010).

19. Westra, H.-J. *et al.* Cell specific eqtl analysis without sorting cells. *PLoS genetics* **11**, e1005223 (2015).

20. Zheng, S. C., Breeze, C. E., Beck, S. & Teschendorff, A. E. Identification of differentially methylated cell types in epigenome-wide association studies. *Nature methods* **15**, 1059 (2018).

21. Mendizabal, I. *et al.* Cell type-specific epigenetic links to schizophrenia risk in the brain. *Genome biology* **20**, 135 (2019).

22. Li, Z., Wu, Z., Jin, P. & Wu, H. Dissecting differential signals in high-throughput data from complex tissues. *Bioinformatics* **35**, 3898–3905 (2019).

23. Rahmani, E. *et al.* Cell-type-specific resolution epigenetics without the need for cell sorting or single-cell biology. *Nature communications* **10**, 1–11 (2019).

24. Luo, X., Yang, C. & Wei, Y. Detection of cell-type-specific risk-cpg sites in epigenome-wide association studies. *Nature communications* **10**, 1–12 (2019).

25. Hansen, L. P. Large sample properties of generalized method of moments estimators. *Econometrica: Journal of the Econometric Society* 1029–1054 (1982).

26. Hansen, L. P., Heaton, J. & Yaron, A. Finite-sample properties of some alternative gmm estimators. *Journal of Business & Economic Statistics* **14**, 262–280 (1996).

27. Jing, H., Zheng, S. C., Breeze, C. E., Beck, S. & Teschendorff, A. E. Calling differential dna methylation at cell-type resolution: an objective status-quo. *BioRxiv* 822940 (2019).

28. Reinius, L. E. *et al.* Differential dna methylation in purified human blood cells: implications for cell lineage and studies on disease susceptibility. *PloS one* **7**, e41361 (2012).

29. Teschendorff, A. E., Breeze, C. E., Zheng, S. C. & Beck, S. A comparison of reference-based algorithms for correcting cell-type heterogeneity in epigenome-wide association studies. *BMC bioinformatics* **18**, 105 (2017).

30. Hannum, G. *et al.* Genome-wide methylation profiles reveal quantitative views of human aging rates. *Molecular cell* **49**, 359–367 (2013).

31. Liu, Y. *et al.* Epigenome-wide association data implicate dna methylation as an intermediary of genetic risk in rheumatoid arthritis. *Nature biotechnology* **31**, 142–147 (2013).

32. Chen, Y.-a. *et al.* Discovery of cross-reactive probes and polymorphic cpgs in the illumina infinium humanmethylation450 microarray. *Epigenetics* **8**, 203–209 (2013).
